# An Erythrocyte‐Templated Iron Single‐Atom Nanozyme for Wound Healing

**DOI:** 10.1002/advs.202307844

**Published:** 2023-12-06

**Authors:** Xiaonan Wang, Ting Liu, Mengxia Chen, Qian Liang, Jing Jiang, Lei Chen, Kelong Fan, Jinhua Zhang, Lizeng Gao

**Affiliations:** ^1^ CAS Engineering Laboratory for Nanozyme Key Laboratory of Biomacromolecules Institute of Biophysics Chinese Academy of Sciences Chaoyang Beijing 100101 China; ^2^ School of Life Sciences University of Chinese Academy of Sciences Haidian Beijing 100049 China; ^3^ College of Life Science and Bioengineering Beijing Jiaotong University Haidian Beijing 100044 China; ^4^ School of Life Science and Technology Jinan University Guangzhou Guangdong 510632 China; ^5^ School of Life Sciences Jilin Normal University Siping Jilin 136000 China; ^6^ Joint Laboratory of Nanozymes in Zhengzhou University Academy of Medical Sciences Zhengzhou University Zhengzhou Henan 450000 China

**Keywords:** antibacterial, erythrocytes, iron single‐atom, nanozyme, peroxidase‐like activity, wound healing

## Abstract

Iron single‐atom nanozymes represent a promising artificial enzyme with superior activity owing to uniform active sites that can precisely mimic active center of nature enzymes. However, current synthetic strategies are hard to guarantee each active site at single‐atom state. In this work, an erythrocyte‐templated strategy by utilizing intrinsic hemin active center of hemoglobin as sing‐atom source for nanozyme formation is developed. By combining cell fixation, porous salinization, and high‐temperature carbonization, erythrocytes are successfully served as uniform templates to synthesize nanozymes with fully single‐atom FeN_4_ active sites which derived from hemin of hemoglobin, resulting in an enhanced peroxidase (POD)‐like activity. Interestingly, the catalytic activity of erythrocyte‐templated nanozyme (ETN) shows dependence on animal species, among which murine ETN performed superior catalytic efficiency. In addition, the as‐prepared ETNs display a honeycomb‐like network structure, serving as a sponge to accelerate hemostasis based on the interactions with prothrombin and fibrinogen. These features enable ETN to effectively kill methicillin‐resistant *Staphylococcus aureus* (MRSA) by combining POD‐like catalysis with near‐infrared (NIR) induced photothermal effect, and subsequently suitable to promote wound healing. This study provides a proof‐of‐concept for facile fabrication of multifunctional nanozymes with uniform single‐atom active sites by utilizing intrinsic iron structure characteristics of biogenic source like erythrocytes.

## Graphical abstract

A novel strategy for synthesizing single‐atom nanozyme with uniform iron active site is developed using erythrocyte as a template containing abundant hemoglobins. The obtained iron single‐atom nanozyme performs antibacterial and hemostatic effects to promote wound healing by combining peroxidase‐like catalysis and honeycomb‐like sponge structure.

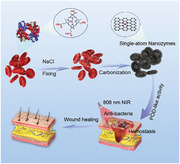

## 1 Introduction

Nanozymes, which are nanomaterials with enzyme­like properties, have been recognized as a new generation of artificial enzymes and bioactive functional nanomaterials with broad applications in biomedical fields.^[^ ^1^ ^]^ Iron‐based nanozymes are a typical representative nanozymes since ferromagnetic (Fe_3_O_4_) nanoparticles were first reported with peroxidase‐like activity,^[^ ^2^ ^]^ showing great potentials in tumor catalytic therapy^[^ ^3^ ^]^ and antimicrobial resistance.^[^ ^4^ ^]^ However, the catalytic efficiency of iron oxide nanoparticles is often lower compared to natural enzymes (e.g., horseradish peroxidase, HRP), as the utilization rate of iron atoms are lower for catalysis.^[^ ^5^ ^]^ To improve this, single‐atom design has been introduced to design precise active sites that can mimic the iron‐porphyrin coordination of natural enzymes, which significantly enhanced the catalytic efficiency of iron and specific activity of nanozymes.^[^ ^6^ ^]^ For instance, the active sites such as FeN_4_, FeN_5_, or FeN_3_P, have been constructed in iron single‐atom nanozymes which perform catalytic efficiency and kinetics matching natural enzymes.^[^ ^7^ ^]^ However, in addition to single‐atom iron, iron clusters or Fe–Fe bonds are also often observed with spherical aberration corrected Transmission Electron Microscope: (ACTEM) or characterized with X‐ray absorption near edge structure (XANES), indicating that the active sites in nanozymes are not uniform in singe‐atom state. This heterogeneity may come from the synthetic methods which often utilize iron salts as the iron source to coordinate with carbonitrides and displace with metals in metal‐organic frameworks (MOFs) followed by a pyrolytic carbonization. For instance, in our previous work, FeCl_3_ was used as iron source to coordinate with dopamine on SiO_2_ nanoparticles, but both sing‐atom Fe and Fe clusters were formed after carbonization at 800 °C.^[^ ^8^ ^]^ In particular, the ratio between single‐atom Fe and Fe clusters was 1:14, indicating that most of iron atoms were not in single‐atom state. The heterogeneity of iron states not only affects the enzyme‐like activity of nanozymes, but also makes it hard to precisely assess their catalytic efficiency.

To solve this problem, we propose an erythrocyte (red blood cell)‐templated strategy using intracellular rich hemoglobin as iron resource to prepare iron single‐atom nanozymes. The basic concept is inspired from the horseradish peroxidase (HRP) with heme as active center comprising a Fe atom coordinated with four N atoms on a same plane.^[^ ^9^ ^]^ Coincidentally, the erythrocytes, primarily tasked with oxygen transport, each contain approximately 260 million hemoglobin molecules,^[^ ^10^ ^]^ each of which contains four iron atoms coordinated with heme plane, making them an ideal source with intrinsic single‐atom iron to make nanozymes that can mimic activity of HRP.^[^ ^11^ ^]^

**Scheme 1:**
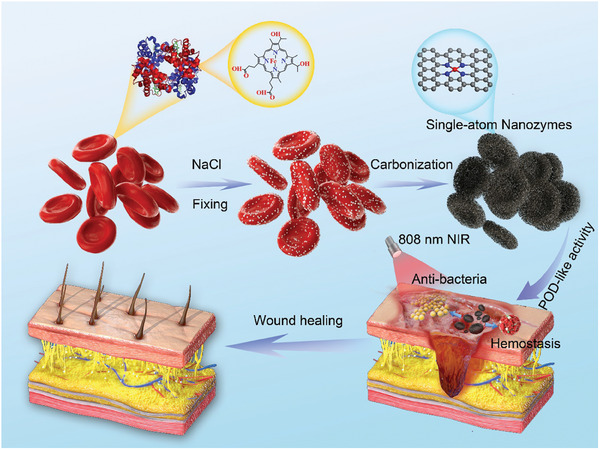
The schematic of using erythrocytes to make single‐atom nanozymes with multifunction properties for wound healing.

To prove our hypothesis, we combined cell fixation, porous salinization, and pyrolytic carbonization to use erythrocytes as both iron and carbon templates to synthesize nanozymes with uniform active site of single‐atom Fe. As depicted in **Scheme** **1**, Such erythrocyte‐templated nanozyme (ETN) exhibited pronounced peroxidase (POD)‐like activity that catalyzes H_2_O_2_ to produce hydroxyl radical (·OH) and can be further enhanced by near‐infrared (NIR) exposure. In addition, the as‐prepared ETNs demonstrated a honeycomb‐like structure, which can be used as a sponge to promote blood coagulation. These features enabled ETNs as a multifunctional material to accelerate wound healing suffering both bleeding and bacterial infection.

## 2 Results and Discussion

### 2.1 Synthesis of Erythrocyte‐Templated Nanozyme

**Figure 1:**
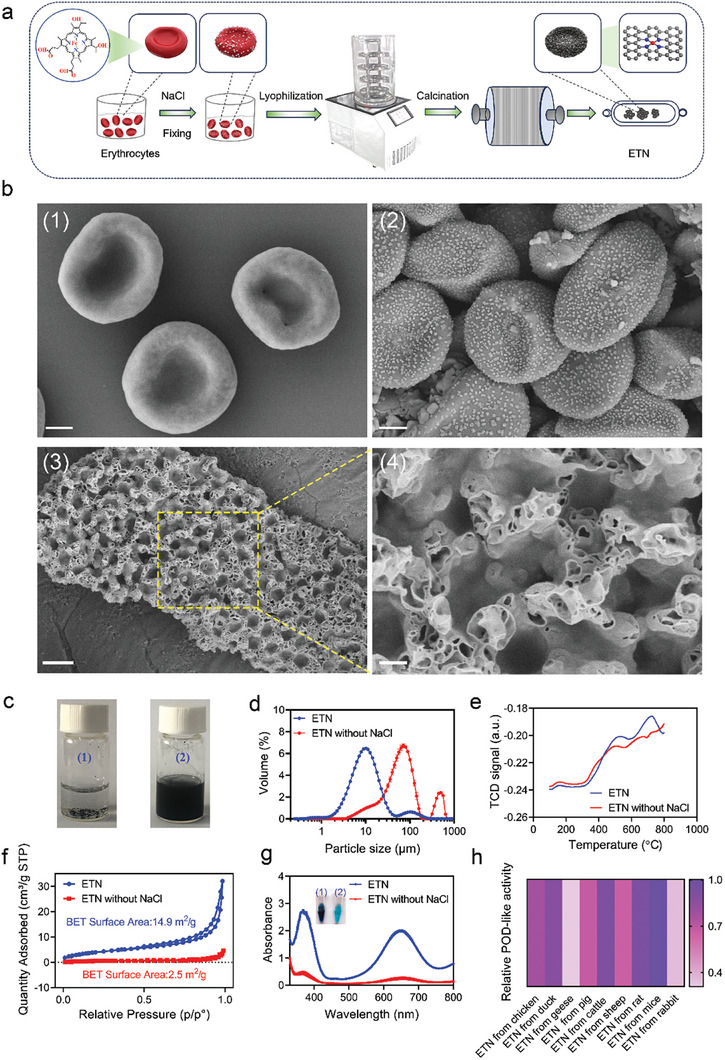
Synthesis of ETN with peroxidase‐like activity. a) A schematic of synthesis process of ETN. b) Scanning electron microcopy of erythrocyte after fixation (1), Salted erythrocyte after lyophilization (2), final carbonized ETN (3). Scale bars = 1 µm (1, 2 and 4) or 5 µm (3). c) Images of the dispersibility in water for ETN (2) and ETN without NaCl (1). d) Dynamic light scattering (DLS) analysis of ETN and ETN without NaCl. e) Temperature‐programmed desorption (TPD) profiles of prepared ETN and ETN without NaCl. f) Brunauer–Emmett–Teller (BET) surface area and N_2_ adsorption–desorption isotherm of ETN and ETN without NaCl. g) Absorption curves of colorimetric reaction for H_2_O_2_ + TMB + ETN and H_2_O_2_ + TMB + ETN without NaCl. Corresponding colors of (1) H_2_O_2_ + TMB + ETN and (2) H_2_O_2_ + TMB + ETN without NaCl are shown. h) Relative POD‐like activity of ETN from nine animal species.

To make soft erythrocyte cell as a suitable template for pyrolytic formation of carbon nanozyme, the cells need to maintain a rigid shape with as less interfering substance as possible (**Figure** **1**a). First, the erythrocytes, abundant in iron‐porphyrins inherent to hemoglobin, were collected from anticoagulant whole blood, fixed with 4% paraformaldehyde (PFA) for 24 h, salinized with 1 m NaCl, and dried using a vacuum freeze dryer (Biocool, China). The ETNs were then obtained after high‐temperature calcination and subsequent washing with ultrapure water. To confirm the influence factors, ETNs were also prepared as controls under different carbonization temperatures from fixed erythrocytes without NaCl treatment. Characterization of the prepared samples was accomplished using scanning electron microscopy (SEM). As shown in Figure 1b, after fixation, the erythrocytes retained their morphology, exhibiting a rounded and biconcave shape (Figure 1b (1); Figure S1a, Supporting Information). Upon drying, numerous salt particles were uniformly dispersed across the erythrocyte surface (Figure 1b (2); Figure S1b, Supporting Information). After carbonization, the ETN exhibited a honeycomb‐like morphology (Figure 1b (3),(4)), whereas the ETN without NaCl displayed an agglomerated spherical shape (Figure S1c, Supporting Information). ETNs showed much better dispersibility in water compared to those without NaCl treatment (Figure 1c). Dynamic light scattering (DLS) ascertained the size of the ETNs with a hydrated particle diameter of 10 µm, whereas that for ETNs without NaCl was up to 72 µm (Figure 1d). Furthermore, Fourier‐transform infrared spectroscopy (FT–IR) showed as evidenced by, a weakened amide bond while diminishing the intensities of both the carboxyl and hydroxyl groups when increasing pyrolytic temperature (Figure S2, Supporting Information), suggesting progressive carbonization of the erythrocyte proteins. The temperature‐programmed desorption (NH_3_‐TPD) profiles were further characterized. As shown in Figure 1e, compared to NaCl‐free ETN with peaks at approximately 150 °C (attributed to the Brønsted acid site) and 500 °C (attributed to the Lewis acid site),^[^ ^12^ ^]^ the ETN exhibited three distinct desorption peaks at approximately 150, 520, and 720 °C, which suggests that ETN has more strong Lewis acid sites that can be positively charged centers to improve catalytic performance.^[^ ^13^ ^]^ Furthermore, Brunauer–Emmett–Teller (BET) analysis showed that the ETN had a higher specific surface area (14.9 m^2^ g^−1^, Figure 1f; Table S1, Supporting Information) than the ETN without NaCl (2.5 m^2^ g^−1^).

Owing to original heme in hemoglobin, ETN was expected to perform enzyme‐like activity similar to HRP which utilizes heme as active center. To prove this, the typical colorimetric reactions were conducted using 3,3′,5,5′‐tetramethylbenzidine (TMB), 2,2′‐azino‐bis (3‐ethylbenzothiazoline‐6‐sulfonic acid) (ABTS), or 1,2‐diaminobenzene (OPD) as chromogenic substrates, combined with the introduction of H_2_O_2_ (Figure S3, Supporting Information). The absorbance value at 652 nm of oxidized TMB (ox‐TMB) catalyzed by the ETN was superior to that catalyzed by the NaCl‐free ETN in the presence of H_2_O_2_ (Figure 1g; Figure S4, Supporting Information), which is consistent with the TPD characteristics of ETN (Figure 1e). Moreover, an increase of POD‐like activity was also observed as the pyrolytic temperature increased and reached the plateau once the temperature over 800 °C (Figure S5, Supporting Information), which is consistent with the FT–IR characteristics (Figure S2, Supporting Information).

To determine the impact of erythrocyte sources on ETN activity, erythrocytes from nine species, including chicken, duck, goose, pig, cow, sheep, rat, mouse, and rabbit were transformed into ETNs following the same procedure (Figure S7, Supporting Information). Interestingly, ETNs derived from chicken, duck, cow, rat, and mouse showed superior POD‐like activity (Figure 1h; Figure S8, Supporting Information), among which mouse‐derived ETNs showed the highest POD‐like catalytic activity at pH 4.5 across a broad temperature range (Figure S9, Supporting Information). To explore what makes such difference, the Fe content of ETNs from different species were detected with inductively coupled plasma optical emission spectrometry (ICP–OES). As shown in Table S2 (Supporting Information), mouse‐derived ETNs showed the highest Fe content, indicating that Fe content has strong correlation with the POD‐like activity of ETNs. Of noted, nonerythrocyte cells, such as Raw264.7 and HaCaT, were also used to make nanozymes, named as RTN (Raw264.7 cells‐templated nanozyme) and HTN (HaCaT cells‐templated nanozyme), respectively. The activity assay showed that neither RTN nor HTN exhibited obvious POD‐like activity (Figure S6, Supporting Information). Consequently, mouse‐derived ETNs were selected as the representative for subsequent study.

### 2.2 Single‐Atom Active Site Characterization of ETN

**Figure 2:**
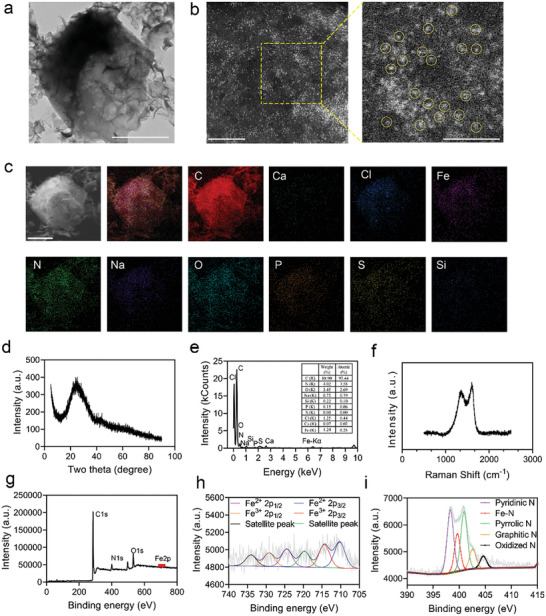
Physicochemical characterization of ETN. a) Transmission electron microscopy (TEM) of ETN. Scale bars = 5 nm. b) Aberration‐corrected high‐angle annular darkfield scanning transmission electron microscopy (AC HAADF‐STEM) of ETN. Scale bars = 5 nm. c) Energy‐dispersive spectrum (EDS) elemental mapping. Scale bars = 1 µm. d) X‐ray diffraction (XRD) peaks of ETN. e) EDS analysis of ETN elements. f) Raman spectra of ETN. g–i) X‐ray photoelectron spectroscopy (XPS) spectra of ETN.

Further characterizations were conducted to determine whether the active site in ETNs is uniform single‐atom iron. Transmission electron microscopy (TEM) depicted a honeycomb network structure (**Figure** **2**a), with a diameter of 1.5 µm. Aberration‐corrected high‐angle annular darkfield scanning transmission electron microscopy (AC HAADF‐STEM) revealed metal species present in the form of isolated bright dots (Figure 2b), which were tagged as single atoms, thus confirming their atomic dispersion on the carbon matrix. Energy‐dispersive spectral (EDS) mapping showed uniform distribution of C, N, O, Fe, Na, and Cl elements across the entire particle, with minimal presence of Si, P, S, and Ca elements (Figure 2c,e). According to ICP–OES, the Fe content in the ETN was 2.47 wt% (Table S3, Supporting Information). The powder XRD pattern of ETN exhibited a broad diffraction around 24°, assigned to the (002) plane of the graphitic carbon structure. The lack of characteristic diffractions of metallic iron and iron oxide implied that the iron species in ETN were atomically dispersed, indicating uniform single‐atom iron (Figure 2d). In addition, Raman spectral analysis showed that the *I*_D_/*I*_G_ value was 0.84, indicating a good graphitization during pyrolytic carbonizations (Figure 2f).

The bound states of N and Fe were also investigated using X‐ray photoelectron spectroscopy (XPS). As shown in Figure 2g–i, the XPS spectra of ETN included C, N, O, and Fe peaks (Figure 2g). The fitted XPS peaks for Fe2p (Figure 2h) at 710.3 (2p3/2) and 724.5 eV (2p1/2) were assigned to Fe^2+^, while the peaks at 714.4 (2p3/2) and 729.2 eV (2p1/2) were assigned to Fe^3+^. The Fe^2+^/Fe^3+^ ratio of 1.2 indicated that ferrous iron was the dominant species in the ETN. Furthermore, the N1s spectra of ETN (Figure 2i) located at 398.3, 399.6, 400.9, 402.6, and 404.6 eV were attributed to pyridinic, Fe–N, pyrrolic, graphitic, and oxidized nitrogen, respectively. The presence of diverse N species provided different chemical/electronic environments for neighboring carbon atoms and hence different catalytic activities.

**Figure 3:**
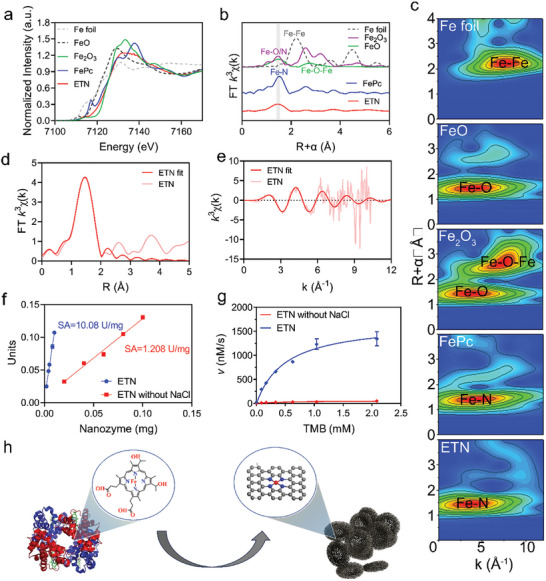
Single‐atom characterization and POD‐like activity of ETN. a) Fe K‐edge XANES spectra. b) Fourier transform analysis of Fe K‐edge EXAFS spectra. c) Wavelet transform analysis of k3‐weighted EXAFS data of ETN and reference samples (FePc, FeO, Fe2O3, and Fe foil). d) R‐space FT EXAFS fitting of ETN. e) k‐space FT EXAFS fitting of ETN. f) Specific activities of ETN and ETN without NaCl. g) POD‐like activity kinetics of ETN and ETN without NaCl at different TMB concentrations. h) A schematic of ETN with FeN_4_ site derived from hemoglobin.

In general, HRP features a single heme as its active site, with an Fe atom as the coordination center and four N atoms as ligands. To determine the atomic arrangement in the as‐prepared ETNs, X‐ray absorption near‐edge structure (XAENS) and extended X‐ray absorption fine structure (EXAFS) spectroscopy were used at the Fe K‐edge. The XANES spectra (**Figure** **3**a; Figure S10, Supporting Information) revealed that the near‐edge absorption energy of the ETNs was located between Fe_2_O_3_ and FePc, demonstrating the presence of positively charged single Fe atoms. The EXAFS curve of ETN showed a primary peak at about 1.5 Å, assigned to the Fe–N scattering paths, but no Fe–Fe peak corresponding to Fe foil at 2.2 Å (Figure 3b). In addition to the R‐space EXAFS spectra, the Fe–O/N and Fe–N scattering pathways were elucidated through wavelet transform (WT) analysis of the ETN EXAFS data (Figure 3c). Subsequent quantitative EXAFS fitting suggested FeN_4_ as the predominant coordination structure of ETN. As shown in Figure 3d,e, the simulated EXAFS spectra of ETN aligned with the experimental data, supporting the FeN_4_ model. The EXAFS fitting parameters (Table S4, Supporting Information) indicated an Fe–N distance at the first coordination shell of 1.98 Å and an average Fe–N coordination of 4.3 ± 0.1. The XANES and EXAFS analyses confirmed the presence of an individual Fe atom in ETN. According to the Fe content detected by ICP, the number of active sites in one ETN was estimated by assuming that one Fe atom represents one active site. The number of active sites was about 2.53 × 10^8^ in each ETN, which is in the same order of magnitude of hemoglobin proteins in a red blood cell (2.60 × 10^8^ per cell). These data indicate that the irons in erythrocyte were highly retained in ETN.

Given the similarity between the active site of FeN_x_ and that of natural HRP (Figure 3h), ETN was anticipated to exhibit comparable POD activity. Colorimetric detection showed that the specific activity of ETN (SA, 10.08 U mg^−1^) was 8.3‐fold higher than that of NaCl‐free ETN (SA, 1.2 U mg^−1^) (Figure 3f). The catalytic efficiency (*k*_cat_/*K*_m_), substrate specificity (*K*_m_), catalytic rate constant (*k*_cat_), and maximal reaction rate (*ν*_max_) for specific substrates, H_2_O_2_ and TMB were further analyzed. According to the obtained catalytic kinetics, ETN had higher binding affinities compared to NaCl‐free ETN for both H_2_O_2_ and TMB substrates. The *k*_cat_/*K*_m_ values for ETN regarding TMB and H_2_O_2_ were 12.8‐ and 44.3‐fold higher, respectively, than those for ETN without NaCl, indicating that NaCl salinization is important to improve catalytic activity (Figure 3g; Figures S11 and S12 and Tables S5 and S6, Supporting Information). Taken together, these physicochemical and catalytic characterizations confirmed that single‐atom iron active sites have been formed and contribute to mimic the active center and activity of natural peroxidase (Figure 3h).

Of noted, besides the POD‐like activity, ETN also showed other enzyme‐like activities as iron is a critical cofactor in many enzymes. As shown in Figure S13 (Supporting Information), the ETN showed multiple enzyme‐like activity. Specifically, the ETN mainly showed oxidase (OXD)‐ and POD‐like activity under acidic condition, superoxide dismutase (SOD)‐, and catalase (CAT)‐like activity under neutral and alkaline conditions. Compared to our previous nanozymes containing single‐atom iron and iron clusters prepared by external iron source,^[^ ^8^ ^]^ ETN showed an increased catalysis in POD‐like activity.

### 2.3 Catalytic Antibacterial Effects and Mechanisms of ETN

**Figure 4:**
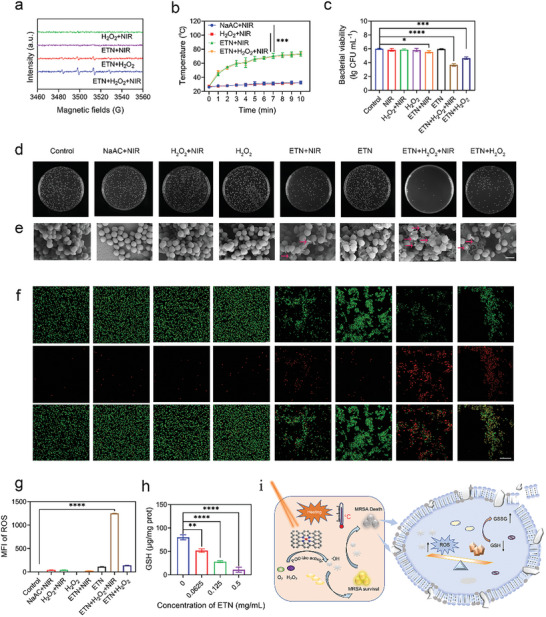
Antibacterial effects and mechanisms of ETN. a) Electron spin resonance (ESR) spectra of DMPO/NaAC solution upon addition of H_2_O_2_ + NIR (2.0 W cm^−2^, 6 min), ETN + NIR, ETN + H_2_O_2_, or ETN + H_2_O_2_ + NIR. b) Temperature‐elevating curves of different treatments. c) Antibacterial effects of ETN with different treatment. d) Agar plate images based on antibacterial effects ETN with different treatment. e) SEM images of MRSA treated with ETN and different treatments. Scale bars = 1 µm. f) Representative CLSM images for a live/dead bacterial viability assay of MRSA with different treatments. Scale bars = 20 µm. g) Reactive oxygen species (ROS) level using DCFH‐DA probe in MRSA treated with ETN and different treatments. h) Glutathione (GSH) levels of MRSA treated with ETN of different concentrations. ^*^ *p* < 0.05. i) The proposed antibacterial mechanism of ETN. ^**^p < 0.01; ^****^p < 0.0001.

The POD‐like activity of ETN is then expected to kill bacteria by mimicking enzyme‐driven ROS generation and caused a glutathione (GSH) depletion for rapid bacteria killing in the phagolysosome of macrophages.^[^ ^14^ ^]^ To prove this, the generation of free ROS was first evaluated via electron spin resonance (ESR) coupled with laser stimulation. The DMPO probe (5,5‐dimethyl‐1‐pyrroline N‐oxide) detected a prominent ·OH signal in the ETN + H_2_O_2_ group, suggesting that the ETNs exhibited POD‐like activity. Notably, the ETN + H_2_O_2_ + NIR group presented higher ·OH intensity, signifying that laser stimulation markedly enhanced the POD‐like activity of the ETN (**Figure** **4**a). This heightened activity is likely due to the ETN reaching its optimal temperature (47.5 °C) for POD‐like activity within a span of 1 min (Figure 4b). Furthermore, ETN displayed a concentration‐dependent temperature elevation (Figure S14, Supporting Information), highlighting its proficiency in converting light to heat. ETN also demonstrated superior thermal stability and photostability, enduring 10 heating and cooling cycles without degradation under sustained laser exposure (Figure S15, Supporting Information). Consequently, the synergistic effects of NIR and the intrinsic POD‐like activity of ETN generate sufficient free ROS, thus establishing a foundation for bacteriostatic applications.

Next, the antibacterial efficacy of ETN against MRSA was assessed using colony forming units (CFUs). As depicted in Figure 4c, when compared to the control only containing NaAc buffer, MRSA counts remained relatively stable in the H_2_O_2_, NIR, ETN, and H_2_O_2_ + NIR groups. In contrast, the ETN + NIR group exhibited an approximately 0.4log reduction of CFU, and ETN + H_2_O_2_ group showed 1.35 log, suggesting that POD‐like activity and the temperature increase partially contributed to sterilization. Notably, the ETN + H_2_O_2_ + NIR group demonstrated significantly enhanced bacteriostatic properties with 2.32‐log reduction of CFU, indicating that the photocatalytic potential of ETN may bolster its bacteriostatic effects in the presence of NIR. These findings were further supported by the bacterial colony counts on plates (Figure 4d). SEM analysis was conducted to study the morphological changes in MRSA in the absence and presence of ETNs with different treatments. For bacterial cells, red arrows indicate different degrees of damage (Figure 4e). Observations revealed that both the untreated and H_2_O_2_‐, NIR‐, H_2_O_2_+ NIR‐, ETN‐exposed MRSA cells maintained a spherical morphology. Posttreatment with ETN + H_2_O_2_ and ETN + NIR manifested evident bacterial damage. Most prominently, ETN + H_2_O_2_ + NIR treatment resulted in notable extrusion of bacterial content. Morphological alterations in bacteria subjected to ETN + H_2_O_2_ + NIR were more pronounced than those exposed to ETN + H_2_O_2_ or ETN + NIR under analogous conditions. These findings aligned with the CFU‐based results, underscoring the superior antibacterial efficacy of ETN + H_2_O_2_ + NIR against MRSA. Further validation was provided by live/dead assays utilizing SYTO‐9 and PI fluorescent staining, with confocal laser scanning microscopy (CLSM) confirming that the ETN + H_2_O_2_ + NIR group exhibited the highest antibacterial activity (Figure 4f).

To elucidate the antibacterial mechanism of the ETN‐based therapeutic platform, ROS production in bacteria after different treatments was evaluated using the fluorescent probe 2′,7′‐dichlorofluorescein diacetate (DCFH‐DA). Results demonstrated pronounced fluorescence intensity in the ETN + H_2_O_2_ + NIR group, indicating higher ROS production (Figure 4g). The levels of GSH, an important antioxidant prevalent in certain bacteria, were also assessed in MRSA under different conditions using the Ellman's assay.^[^ ^15^ ^]^ As depicted in Figure 4h and Figure S16 (Supporting Information), GSH levels in MRSA were markedly reduced after ETN + H_2_O_2_ + NIR treatment, while no discernible changes were observed in the ETN + NIR or ETN + H_2_O_2_ groups. This suggests that ETN caused a GSH depletion due to ROS generation and then the disruption of the intrinsic balance in the bacterial microenvironment (Figure 4i). Notably, this effect was magnified by NIR exposure, which precipitated bacterial death in a concentration‐dependent manner. These results suggest that ETN may kill bacteria by inducing ferroptosis‐like death which has been proved in many other antibacterial nanozymes.^[^ ^16^ ^]^ Of noted, such antibacterial mechanism of action is not specific. In addition to Gram‐positive *Staphylococcus aureus*, ETN also showed considerable antibacterial effect on Gram‐negative *Escherichia coli* (Figure S17, Supporting Information), which is similar to that of MRSA (Figure 4c), indicating that the ETN has a broad antibacterial spectrum.

### 2.4 Procoagulant Effects of ETN Sponge

**Figure 5:**
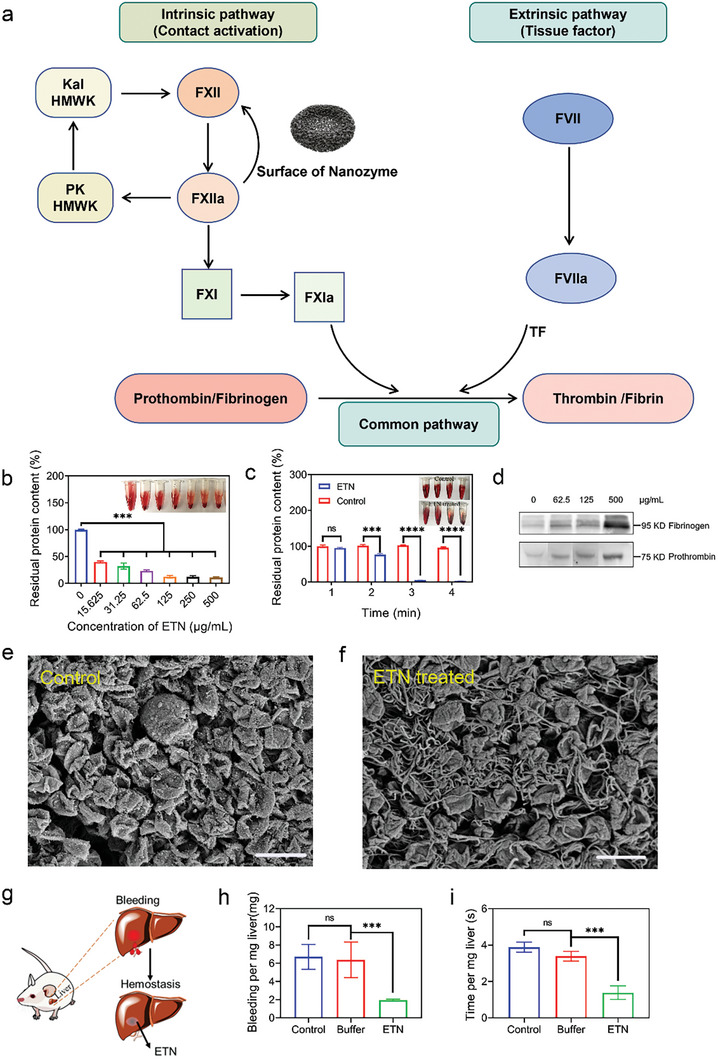
Procoagulant effects and molecular mechanisms of ETN. a) A schematic of coagulation cascade reaction. Intrinsic pathway: Surface‐mediated interactions in contact activation of plasma coagulation involve PK, HMWK, FXII, FXI, and Kal. Suffix “a” represents activated proteins. FXII “binds” to a negatively charged surface (represented nanozymes), inducing conformational change and transformation into the active‐enzyme form FXIIa through autoactivation.^[^ ^23^ ^]^ In turn, surface‐generated FXIIa can cleave surface‐bound PK complexed with HMWK, then activate surface‐bound FXI complexed with HMWK to generate FXIa, leading to propagation of subsequent coagulation cascade reactions. Extrinsic pathway: tissue factor (TF) produced during trauma induces FVIIa formation. These two pathways lead to the common pathway producing thrombin or fibrin.^[^ ^24^ ^]^ b) Procoagulant effects of different concentrations of ETN in vitro. Insets show procoagulant effects of different concentrations of ETN in vitro. Results are given as percentage of protein in the supernatant relative to the control sample without ETN. c) Procoagulant effects of ETN at different times. Insets show procoagulant effects of ETN at different times. d) Western blot analysis of fibrinogen and prothrombin in blood treated by ETN. e,f) SEM of blood from control (e) and ETN‐treated groups (f). Scale bars = 5 µm. g) Schematic of procoagulant in vivo. h,i) Procoagulant effects of ETN in vivo. ^**^ *p* < 0.01; ^***^ *p* < 0.001.

Beside catalytic properties, we speculate that another feature, the honeycomb‐like structure, may endow ETN to act as a sponge for fast biomass adsorption. This special property is essential for wound dressing materials, such as hydrogels,^[^ ^17^ ^]^ cellulose sponges,^[^ ^18^ ^]^ and nanofibers.^[^ ^19^ ^]^ For instance, cellulose sponges show outstanding hemostatic effect due to their distinct 3D porous structure composed of high‐aspect ratio nanofibers. With a similar 3D porous network structure, ETN may also perform as potential a hemostatic agent predominantly participating in the intrinsic coagulation cascade pathway (**Figure** **5**a).^[^ ^20^ ^]^ To prove this, the hemostatic efficacy of ETN was firstly evaluated using blood clotting tests.^[^ ^21^ ^]^ In the control group without ETN, the samples after water rinsing turned red due to the presence of uncoagulated blood, suggesting ineffective clotting in the absence of a hemostatic agent. In contrast, an increase in blood clot formation was observed when ETN concentration was increased gradually (Figure 5b). Notably, blood agglutination occurred within 3 min in the presence of 50 µg mL^−1^ ETN (Figure 5c), indicating that ETNs promoted blood clotting. Western blot analysis was further applied to examine fibrinogen and prothrombin interactions with ETN. Results showed that fibrinogen and prothrombin content in ETNs increased with increasing concentration (Figure 5d), indicating that ETNs can directly adsorb fibrinogen and prothrombin, as confirmed by SEM (Figure 5e,f).

A significant proportion of trauma‐associated fatalities arise from uncontrolled bleeding, especially hemorrhage from noncompressible wounds.^[^ ^22^ ^]^ Here, the hemostatic nature of the as‐produced ETN was tested using a mouse liver defect wound model to simulate uncontrollable bleeding (as depicted in Figure 5g). In the model, a segment of liver tissue was excised, with a cotton ball saturated with buffer or ETN then applied to compress the wound until bleeding stopped. A segment of liver was also removed without further treatment as a control. Various parameters, including bleeding amount, bleeding duration, and weight of removed liver, were recorded. Bleeding amount and bleeding time per mg of liver were then calculated to evaluate the hemostatic properties of the ETNs. Compared with the control group, the ETN‐treated group exhibited a marked reduction in bleeding amount (Figure 5h) and clotting time (Figure 5i), demonstrating the potential of ETN as a hemostatic agent.

### 2.5 Promoting Wound Healing with ETN

**Figure 6:**
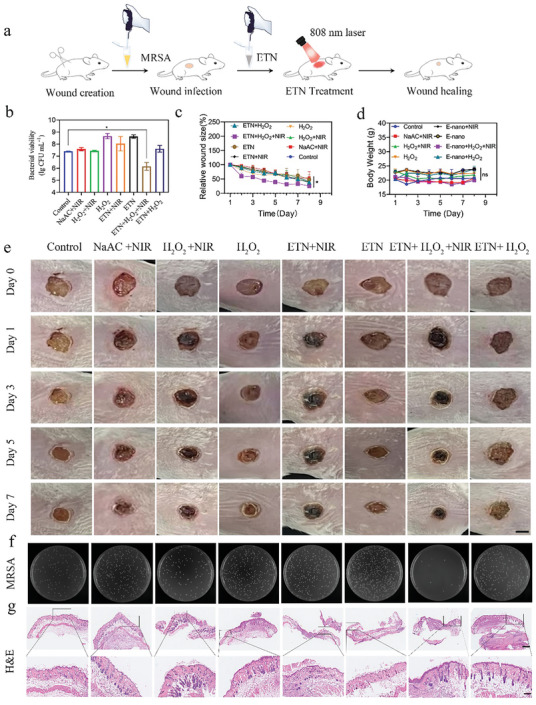
Promoting wound healing by ETN in animal model infected with MRSA. Eight‐week‐old Balb/c female mouse groups (n = 6 per group) were subjected to wound infection. Data are representative of at least three independent experiments. Eight groups were: 1) Control, 2) NaAC + NIR, 3) H2O2 + NIR, 4) H_2_O_2_, 5) ETN + NIR, 6) ETN, 7) ETN + H_2_O_2_ + NIR, and 8) ETN + H_2_O_2_, treated with 100 µm H_2_O_2_ and 500 µg mL^−1^ ETN, respectively. a) The procedure for infected wound animal model establishment and treatment with ETN. b) Statistical results of bacterial survival in mouse wounds. c,d) Relative wound size and body weight of mice in different groups. e) Time‐dependent photographs of wounds in mice under different treatments. Scale bars = 4 mm. f) Agar plate images of antibacterial effects under different treatments for 7 days. g) H&E staining of wound tissues in different groups. ^*^ *p* < 0.05. Scale bars = 10 µm (up) and 200 µm (down).

To further assess the potential of ETN for wound healing, we developed an injury‐infection animal model to evaluate the in vivo therapeutic effects.^[^ ^25^ ^]^ In brief, a 100‐mm^2^ wound was created on the back of Balb/c mice, which was subsequently inoculated with MRSA at a concentration of 5 × 10^7^ CFU per wound (**Figure** **6**a). In vitro experiments showed that the ETNs exhibited enhanced antibacterial activity against MRSA by combining POD‐like activity and NIR‐mediated hyperthermia (Figure 4c,e–g). Thus, for in vivo study, the mice were divided into eight groups: 1) 0.2 m NaAc (Control), 2) NaAc + NIR, 3) H_2_O_2_ (100 µm) + NIR (2.0 W cm^−2^, 6 min), 4) H_2_O_2_, 5) ETN + NIR, 6) ETN, 7) ETN + H_2_O_2_ + NIR, and 8) ETN + H_2_O_2_. To mitigate skin burns, the buffer was refreshed every 2 min, with the NIR exposure dose set to 2.0 W cm^−2^ for 6 min. The total volume of administered ETN (500 µg mL^−1^) was 20 µL. After topical MRSA infection, the images of the wounds were taken (Figure 6e). A distinct yellow color at the wound site confirmed the successful establishment of the infection model. Posttreatment, no significant changes in mouse weights were observed, indicating the absence of adverse reactions to the infection (Figure 6d). Bacterial counts of the wound sites were evaluated for each group on day 7. As shown in Figure 6b,f, the CFU values in the ETN + H_2_O_2_ + NIR group were significantly reduced. The quantitative analysis of wound areas for each group is depicted in Figure 6c and Figure S18 (Supporting Information). Specifically, after 3 days of treatment, the residual wound areas for groups 1 to 8 were 42%, 54%, 41%, 38%, 39%, 48%, 26%, and 38%, respectively. These findings highlight the combined role of hyperthermia and the POD activity of ETN in facilitating enhanced wound healing. The wound tissues were then observed by hematoxylin and eosin (H&E) staining on day 7 (Figure 6g). While inflammatory cells persisted in the control and other groups, the ETN + H_2_O_2_ + NIR group displayed a prominent presence of fibroblasts and a continuous, intact skin tissue structure, indicating promoted wound recovery. Collectively, these findings suggest that ETNs hold considerable promise as a multifunctional material for would healing by preventing infection and rapid hemostasis.

The cytotoxicity of ETN was also assessed in vitro. HaCaT (Figure S19a, Supporting Information) and Raw264.7 (Figure S19b, Supporting Information) cells exposed to ETN for 24 h demonstrated negligible decline in cellular viability, even at an ETN concentration up to 250 µg mL^−1^. Furthermore, as shown in Figure S20 (Supporting Information), biocompatibility assessments indicated that while the positive control exhibited evident hemolysis, no hemolytic events were associated with ETN at any concentration. Histological evaluations of primary organs, including the heart, liver, spleen, lung, and kidney, from the wound healing experiments demonstrated no notable pathological changes (Figure S21, Supporting Information), indicating a high histocompatibility of ETN.

## 3 Conclusion

In summary, we report a new strategy to develop iron single‐atom nanozymes by utilizing erythrocyte containing intrinsic heme irons from high content hemoglobin. We demonstrate that erythrocyte can be used as a template to prepare nanozymes by combining cell fixation, porous salinization, and pyrolytic carbonization. The as‐prepared erythrocyte‐templated nanozymes (ETNs) possess uniform FeN_4_ single‐atom active site which can perform peroxidase‐like activity. Interestingly, the catalytic activity of ETNs showed a correlation with animal species, as a big difference was observed between mouse and geese. We demonstrate that the ETNs can be used to kill resistant bacteria such as MRSA upon their peroxidase‐like activity which can be enhanced by NIR‐mediated photothermal effect. Furthermore, the honeycomb‐like structure makes ETNs act as a sponge to promote hemostasis by inducing coagulation reaction. The proof‐of‐concept animal experiments have validated the application potential of ETN as a multifunctional material in wound healing.

## Conflict of Interest

The authors declare no conflict of interest.

## Supporting information

Supporting Information

## Data Availability

The data that support the findings of this study are available from the corresponding author upon reasonable request.
