# Supplementary material for: An Erythrocyte‐Templated Iron Single‐Atom Nanozyme for Wound Healing
Source: Adv Sci (Weinh). 2023 Dec 6;11(6):2307844. doi: 10.1002/advs.202307844 (PMC10853745; doi:10.1002/advs.202307844)
Supplement: Supplementary file 1 — Supporting Information [file ADVS-11-2307844-s001.pdf]

ADVANCED SCIENCE
Open Access

Supporting Information

for *Adv. Sci.*, DOI 10.1002/advs.202307844

An Erythrocyte-Templated Iron Single-Atom Nanozyme for Wound Healing

Xiaonan Wang, Ting Liu, Mengxia Chen, Qian Liang, Jing Jiang, Lei Chen, Kelong Fan, Jinhua Zhang and Lizeng Gao**

Supporting Information

An erythrocyte-templated iron single-atom nanozyme for wound healing

Xiaonan Wang[1,2,†], Ting Liu[3,4,†], Mengxia Chen[3,5], Qian Liang[1], Jing Jiang[1], Lei Chen[1], Kelong Fan[1,6], Jinhua Zhang[3], Lizeng Gao[1,6*]*

X.N. Wang, Q. Liang, J. Jiang, L. Chen, Prof. K.L. Fan, Prof. L.Z. Gao
[1]CAS Engineering Laboratory for Nanozyme, Key Laboratory of Protein and Peptide Pharmaceutical, Institute of Biophysics, Chinese Academy of Sciences, Chaoyang, Beijing 100101, China
[2]School of Life Sciences, University of Chinese Academy of Sciences, Haidian, Beijing 100049, China
Email: gaolizeng@ibp.ac.cn (L.G.)

T. Liu, M.X. Chen, Prof. J.H. Zhang
[3]College of Life Science and Bioengineering, Beijing Jiaotong University, Haidian, Beijing 100044, China
[4]School of Life Science and Technology, Jinan University, Guangzhou, Guangdong 510632, China
[5]School of Life Sciences, Jilin Normal University, Siping, Jilin 136000, China
Email: _zhangjh@bjtu.edu.cn_(J.Z.)

Prof. K.L. Fan, Prof. L.Z. Gao,
[6]Joint Laboratory of Nanozymes in Zhengzhou University, Academy of Medical Sciences, Zhengzhou University, Zhengzhou, Henan 450000, China

*Correspondence authors.
†These authors contributed equally to this work.

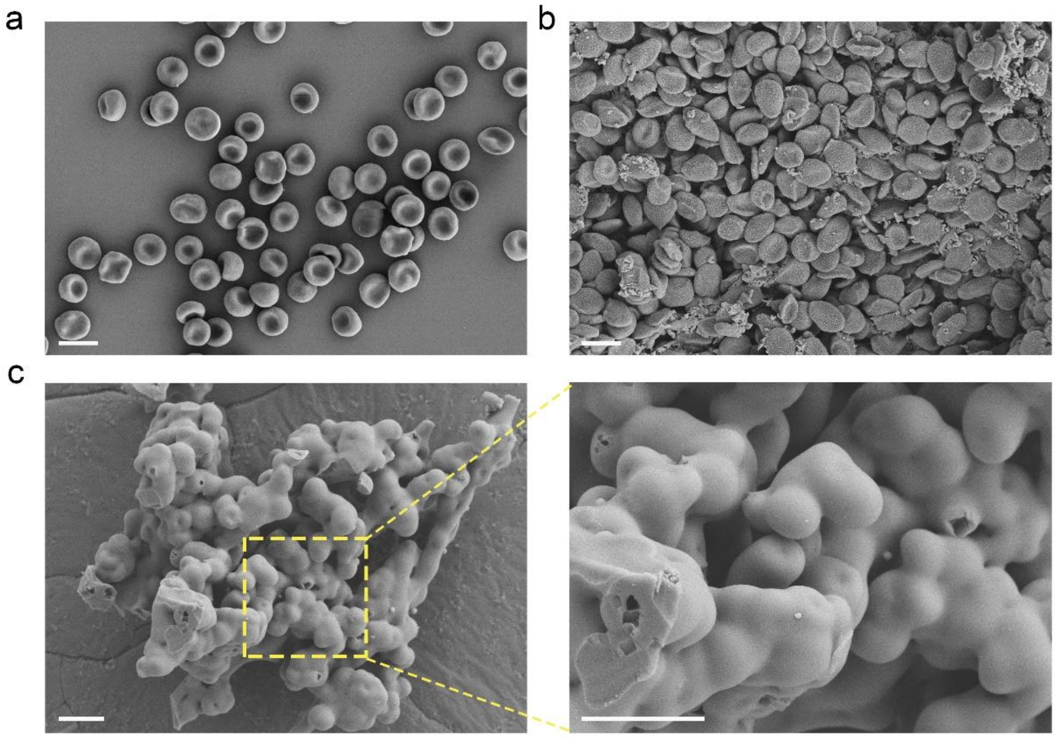

Figure S1. Characterization of the Erythrocyte-templated nanozyme (ETN) preparation process. a, Erythrocytes after fixation. b, Salted erythrocytes prepared by lyophilization. c, The final obtained ETN without NaCl. Scale bars: 5 μm.

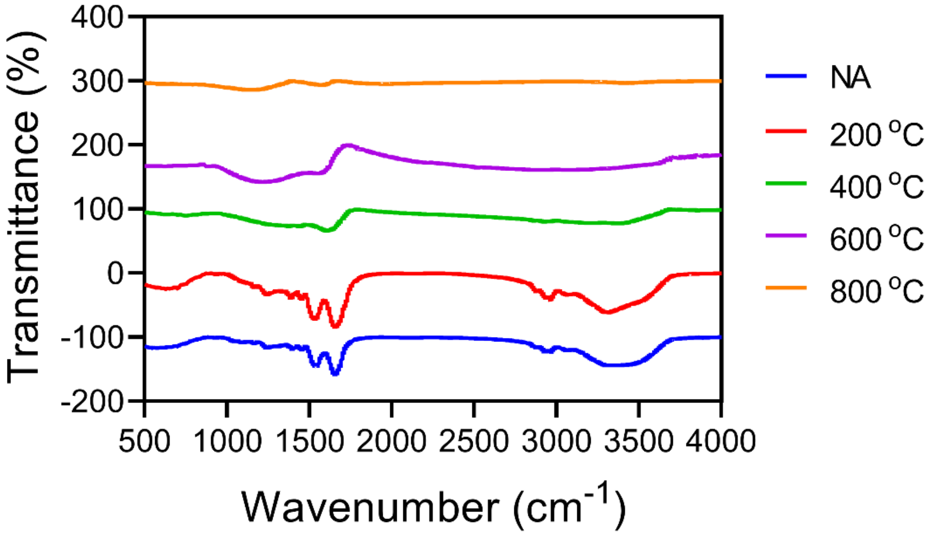

Figure S2. FT-IR characterization of ETNs by different carbonation temperature.

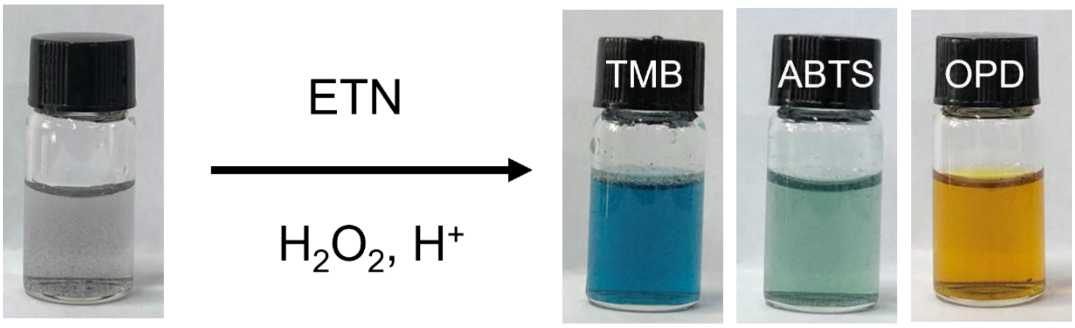

Figure S3. The ETN catalysis oxidation of various peroxidase substrates in the presence of H_2O_2 to produce different color reactions.

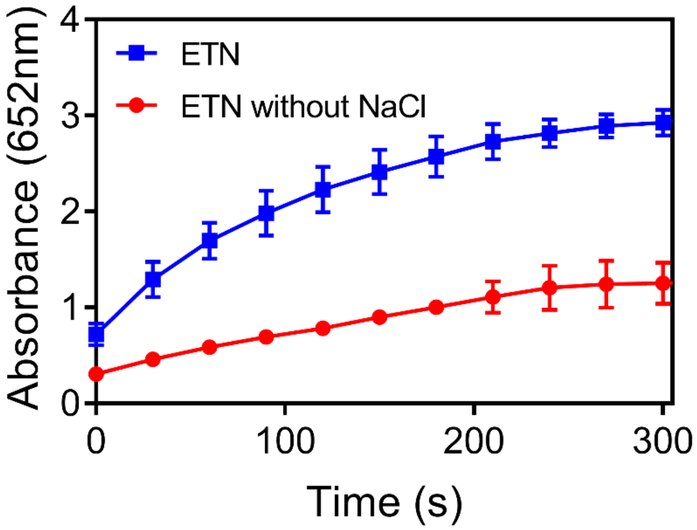

Figure S4. Time-dependent absorbance of TMB/H_2O_2/ETN or TMB/H_2O_2/ETN without NaCl.

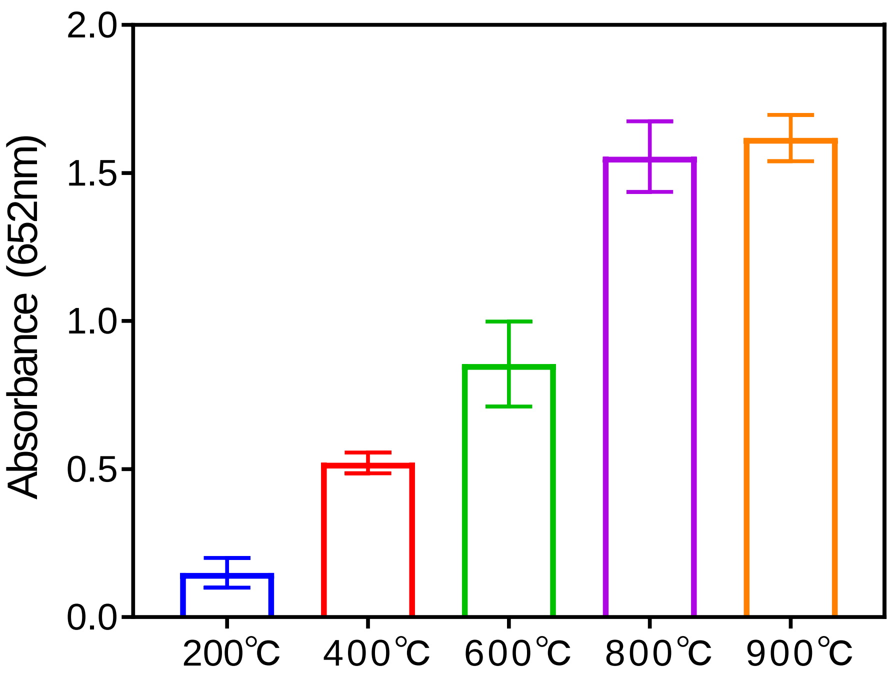

Figure S5. The assessment on the catalytic performance of ETNs prepared under different carbonation temperature. The catalytic reaction of peroxidase-like activity was conducted with TMB substrate.

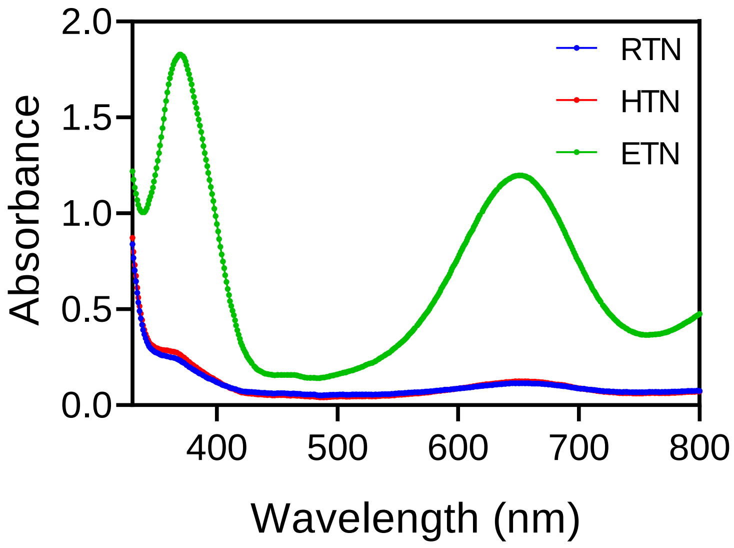

Figure S6. The comparison of peroxidase-like catalysis between ETN and other cells-derived nanozymes including RTN (Raw264.7-templated nanozyme) and HTN (HaCaT-templated nanozyme).

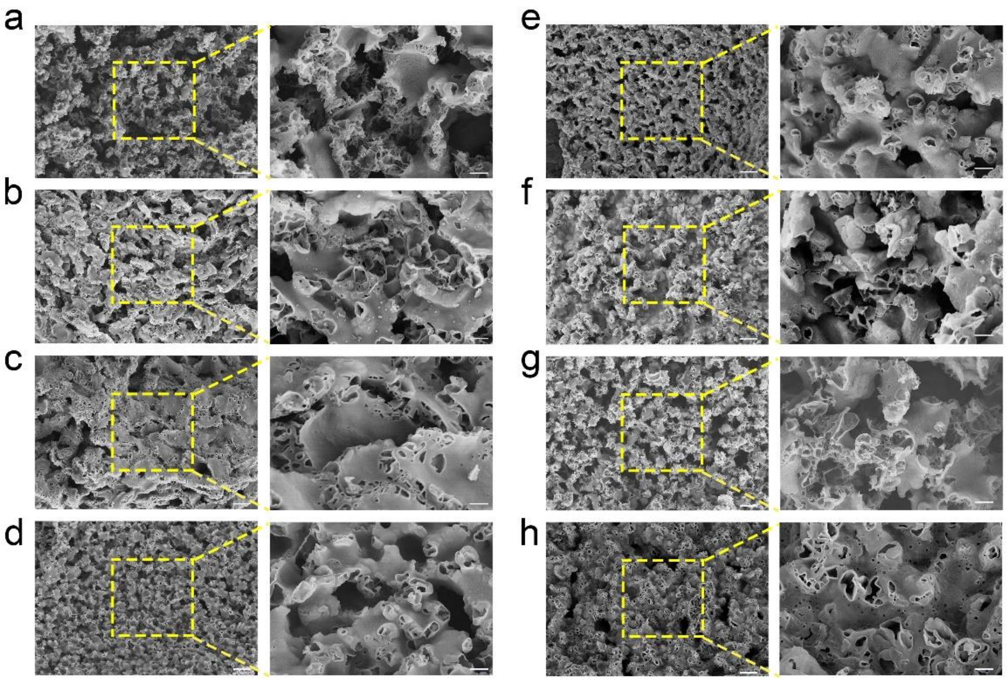

Figure S7. ETNs derived from different species. a, ETN derived from chicken. b, ETN derived from duck. c, ETN derived from goose. d, ETN derived from pig. e, ETN derived from cow. f, ETN derived from sheep. g, ETN derived from rat. h, ETN derived from rabbit. Scale bars: 5 μm (left) and 1 μm (right).

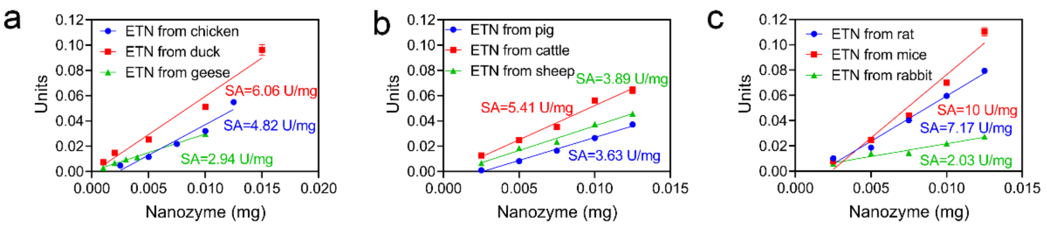

Figure S8. The specific POD-like activities (U/mg) of ETN from nine different species. a, POD-like activities of ETN derived from chicken, duck or geese. b, POD-like activities of ETN derived from pig, cattle or sheep. c, POD-like activities of ETN derived from rat, mice or rabbit. The nanozyme activity (U) is defined as the amount of nanozyme that converts 1 micromole of substrate per minute.

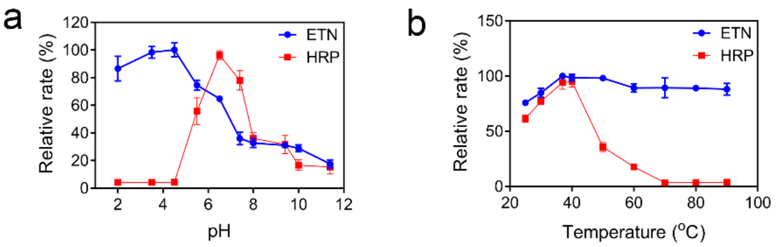

Figure S9. The peroxidase-like activity of the ETN is pH dependent (a) but showed high catalytic activity in a wide temperature range (b) using TMB as the substrate.

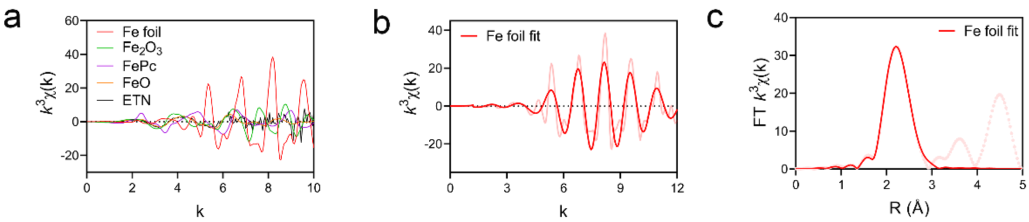

Figure S10. EXAFS spectra of ETN and Fe foil. a, Fourier transform of Fe K-edge EXAFS spectra. b, The FT EXAFS fitting of Fe foil at the k-space and c, The FT EXAFS fitting of Fe foil at the R-space.

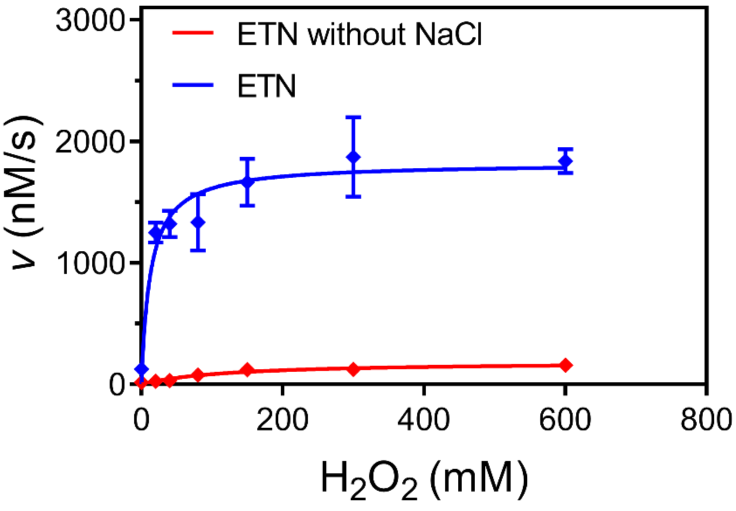

Figure S11. Kinetics for POD-like activity of ETN, ETN without NaCl, at different H_2O_2 concentration.

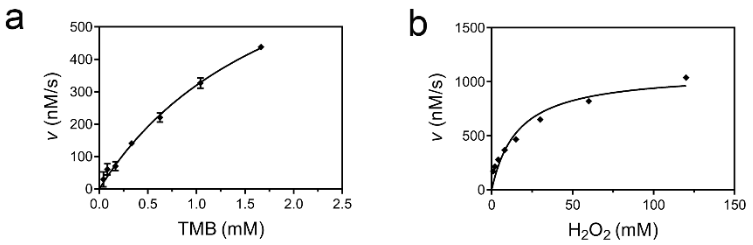

Figure S12. Kinetics for POD activity of HRP at different (a) TMB or (b) H_2O_2 concentration.

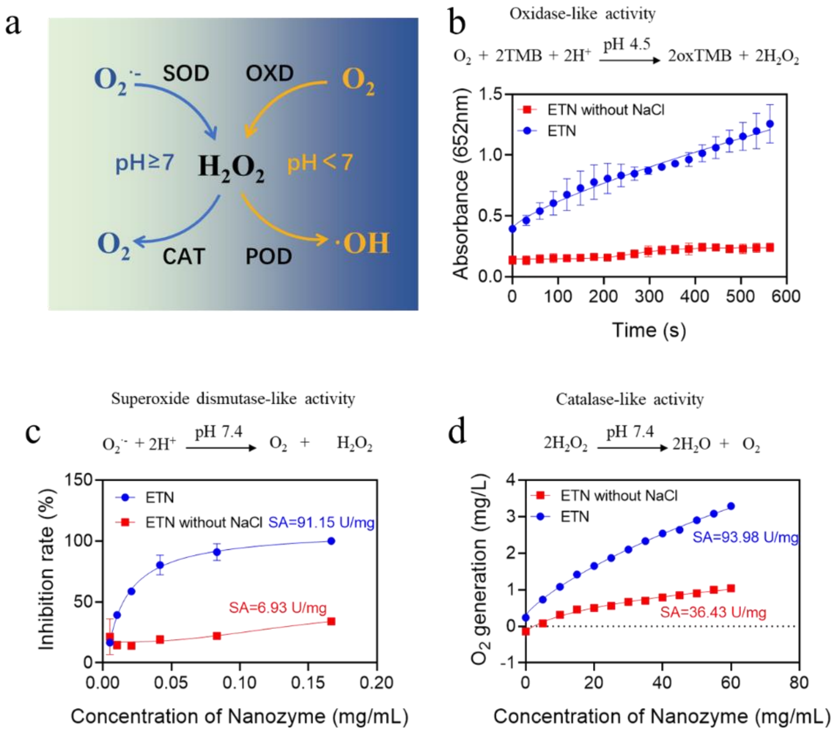

Figure S13. Other enzyme-like activities of ETNs. a, A scheme illustration of the multiple enzyme-like activity of ETN. b, Oxidase-like activity of ETN. c, Superoxide dismutase-like activity of ETN. d, Catalase-like activity of ETN.

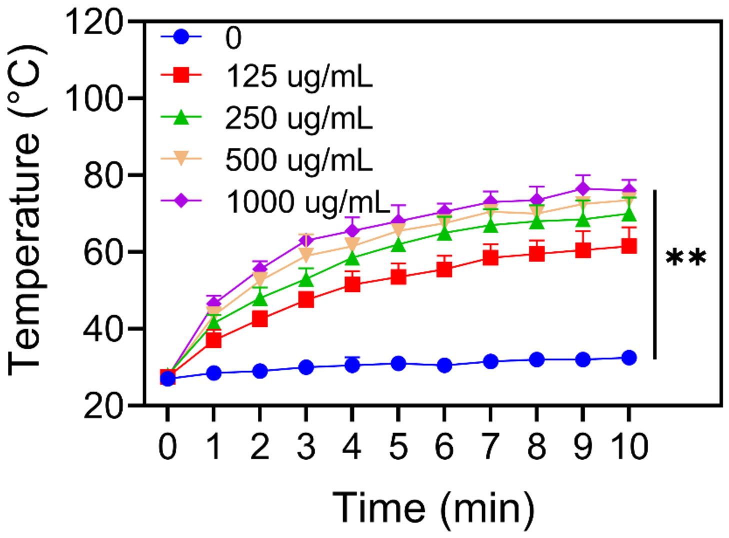

Figure S14. Temperature changes of different concentration ETN on NIR 808 nm laser irradiation.

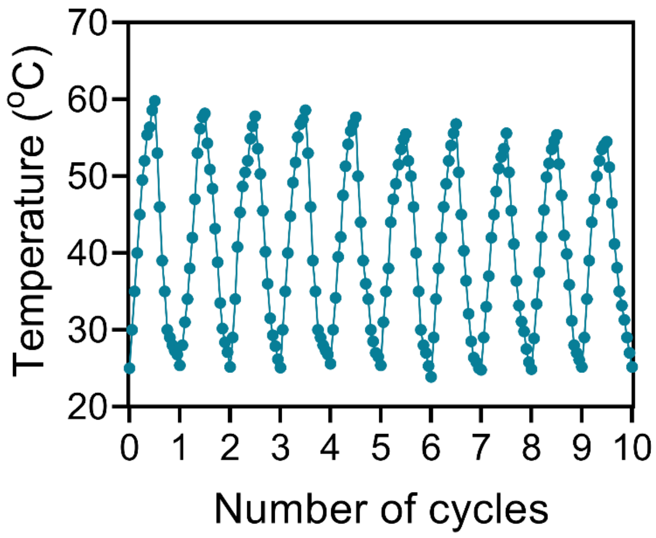

Figure S15. Photothermal stability study of ETN during ten circles of heating-cooling processes.

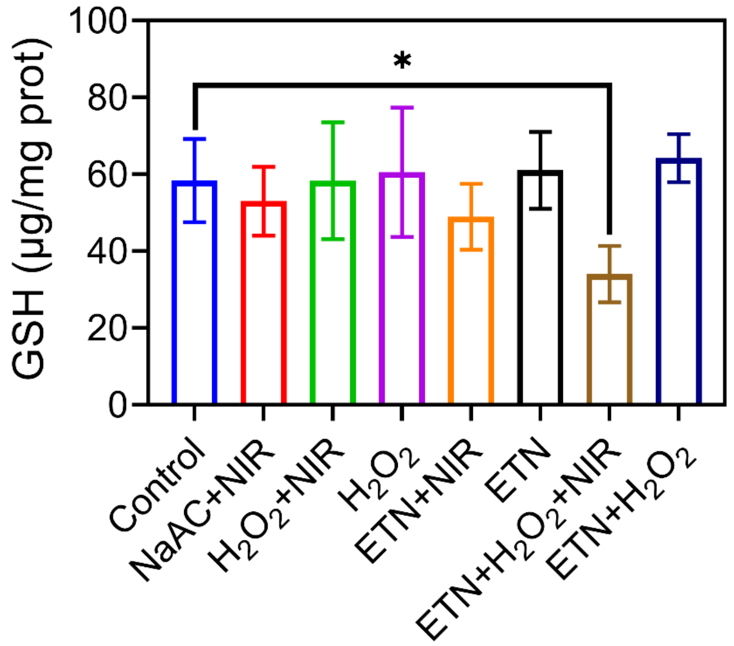

Figure S16. The GSH levels of MRSA treated with different condition.

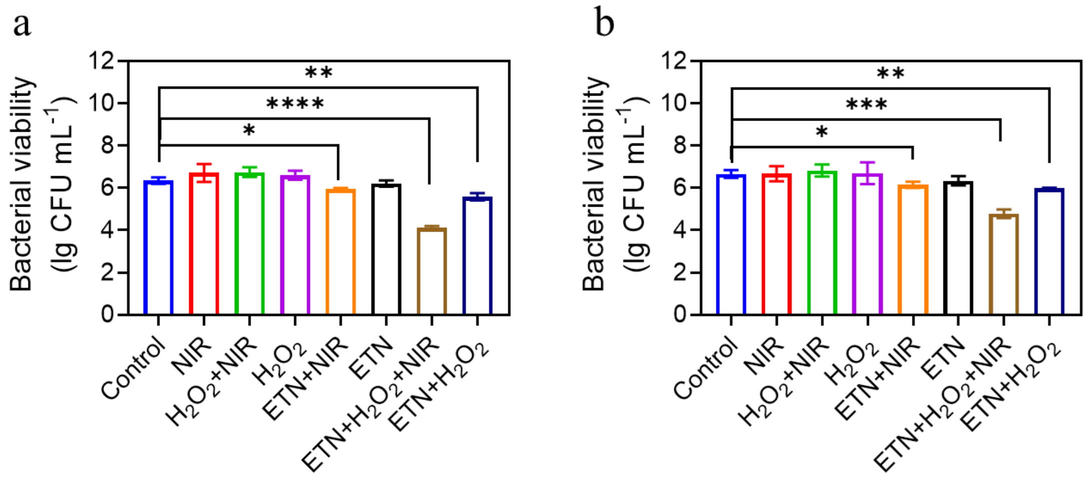

Figure S17. Antibacterial effects to different bacterial species. a, *E. coli* and b, *S. aureus* of ETN with different treatment.

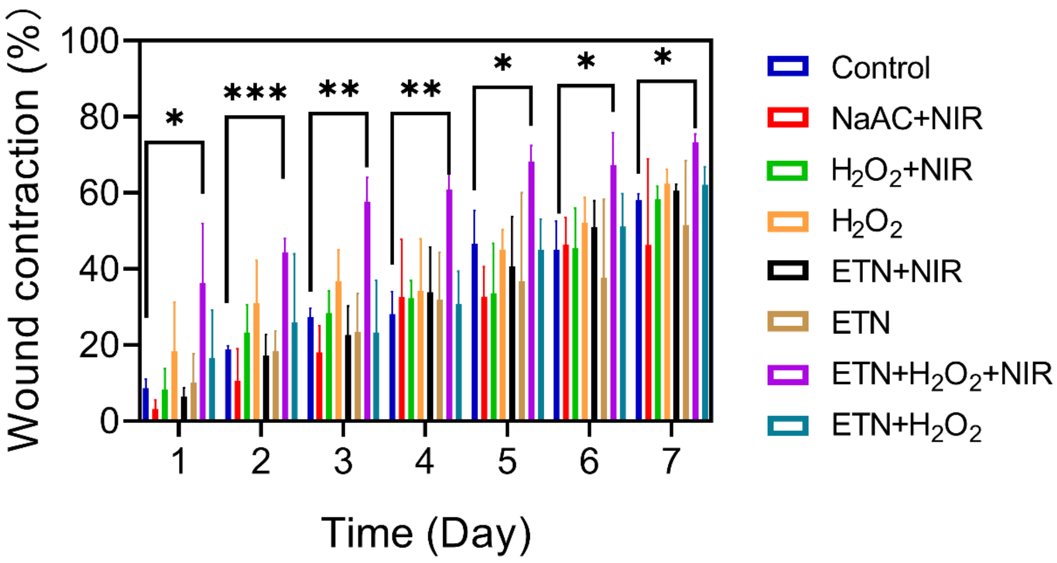

Figure S18. Wound contraction (%) changes of mice after different treatments.

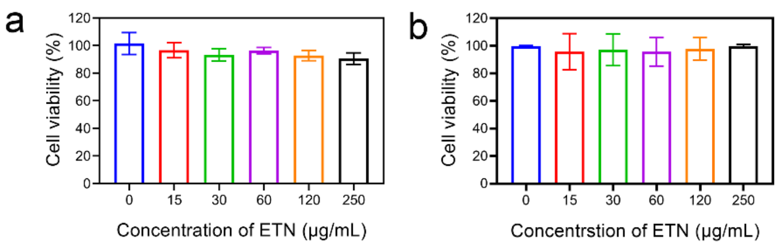

Figure S19. Cytotoxicity of ETN was determined by CCK-8. a, HaCaT. b, Raw 264.7.

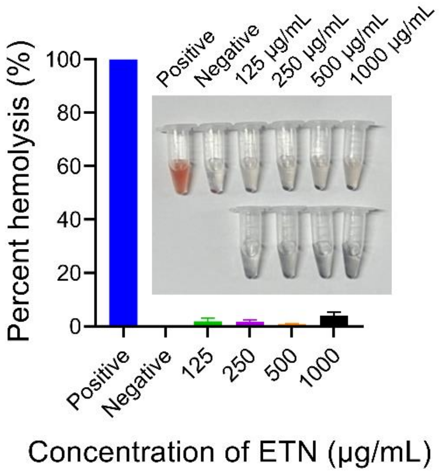

Figure S20. Relative hemolysis ratio of different concentrations of ETN (ranging from 125 to 1000 μg mL^{-1}).

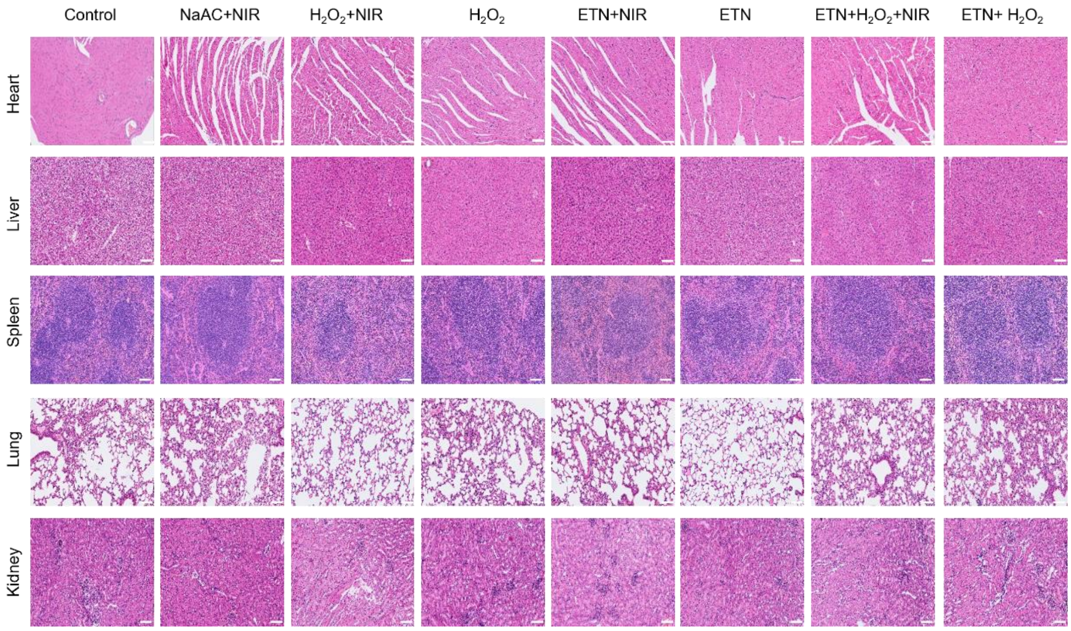

Figure S21. H&E staining photographs of main organs from the mice with different treatments (7 days). Scale bars = 100 μm.

Table S1. BET and pore volume of ETN and ETN without NaCl.

Nanozymes	BET (m^2/g)	Pore Volume (cm^3/g)
ETN	14.9071	0.0497
ETN without NaCl	2.5087	0.0072

Table S2. Fe content of ETN from different species.

ETN	Fe content (wt.%)
ETN from chicken	0.9430%
ETN from duck	1.2100%
ETN from geese	0.8480%
ETN from pig	0.8728%
ETN from cattle	1.1227%
ETN from sheep	0.9028%
ETN from rat	1.6275%
ETN from mice	1.7254%
ETN from rabbit	0.8101%

Table S3. Elements content of ETN and ETN without NaCl.

Elements	ETN Without NaCl (Wt.%)	ETN (Wt.%)
Ca	0.0331	0.0179
Na	1.5951	0.5347
P	0.7065	0.6334
S	0.1840	0.3705
Si	0.7711	0.2171
Fe	1.2026	2.4747

Table S4. EXAFS fitting parameters at the Fe K-edge for various samples (S_0^2 = 0.81).

	shell	CN	R(Å)	σ^2	ΔE_0	R factor
Fe foil	Fe-Fe1	8	2.47 ± 0.01	0.0047	-4.2 ± 0.8	0.0037
	Fe-Fe2	6	2.85 ± 0.01	0.0060		
Sample	Fe-N	4.3 ± 0.1	1.98 ± 0.01	0.0106	-1.0 ± 0.5	0.0002

[a]*CN*: coordination numbers; [b]*R*: bond distance; [c]σ^2: Debye-Waller factors; [d] ΔE_0: the inner potential correction. *R* factor: goodness of fit.

Table S5. Comparison of the kinetics for the substrate TMB based on Fe active sites doped on ETN, ETN without NaCl or HRP enzyme. [E/Fe] is the molar concentration of the Fe active sites of nanozymes or HRP enzyme, which was chosen to obtain the well-fitted Michaelis-Menten plots while varying the substrate concentrations. K_m is the Michaelis constant, v_{max} is the maximal reaction velocity and k_{cat} is the catalytic constant, where k_{cat} = v_{max}/[E/Fe] and the k_{cat}/K_m value indicates the catalytic efficiency of the enzyme or nanozyme.

	[E/Fe] (M)	K_m (M)	v_{max} (M s^{-1})	k_{cat} (s^{-1})	k_{cat} /K_m ($M^{-1}s^{-1}$)
ETN	8.02×10^{-7}	0.5×10^{-3}	1.7×10^{-6}	2.12	4.24×10^{3}
ETN without NaCl	3.9×10^{-7}	0.43×10^{-3}	5.5×10^{-8}	0.14	3.3×10^{2}
HRP	2.5×10^{-12}	4.6912×10^{-7}	3.1628×10^{-8}	1.27×10^{4}	2.70×10^{10}

Table S6. Kinetics parameters of ETN, ETN without NaCl or HRP enzyme for H_2O_2 substrate. [E/Fe] is the molar concentration of the Fe active sites doped on nanozymes, K_m is the Michaelis constant, v_{max} is the maximal reaction velocity and k_{cat} is the catalytic constant, where k_{cat} = v_{max}/[E/Fe] and the k_{cat}/K_m value indicates the catalytic efficiency of the enzyme or nanozymes. (n = 3 independent measurements, bars represent means ± SD).

	[E/Fe] (M)	K_m (M)	v_{max} (M s^{-1})	k_{cat} (s^{-1})	k_{cat} /K_m ($M^{-1}s^{-1}$)
ETN	8.02×10^{-7}	1.28×10^{-2}	1.8×10^{-6}	2.27	1.77×10^{2}

ETN without NaCl	3.9×10^{-7}	1.2×10^{-1}	1.88×10^{-7}	4.8×10^{-1}	4
HRP	2.5×10^{-12}	4.26723	2.57515×10^{-7}	1.03×10^{4}	2.41×10^{6}

Materials and methods

Structural characterization.

The ETNs in deionized water were embedded in double-sided copper grid (Zhongjingkeyi Films Technology Co., Ltd.) pretreated by current and imaged with transmission electron microscope (TEM) (TETNai Spirit-120kV, FEI, USA). The diameter of ETNs (0.5 mg mL^{-1}) in deionized water were characterized by the dynamic light scattering (DLS) with DynaPro Titan (Wyatt Technology, USA). X-ray photoelectron spectroscopy (XPS) (Thermo Scientific ESCALAB 250, USA) was used to characterize the elemental composition and chemical state of ETNs. Scanning electron microscope (SEM) (Hitachi SU8010, Japan) was used to characterize the morphology of ETN and morphological changes of bacteria. The Electron paramagnetic resonance (EPR) spectroscopy was measured by a Variable temperature electron paramagnetic resonance spectrometer E500 (Bruke, German). The Raman spectra were obtained at a Renishaw 1000 microRaman system. The aberration-corrected high-angle annular dark-field scanning transmission electron microscopy (AC-HAADF-STEM) was performed by a JEM-ARM200F (JEOL, Japan). All the UV-vis spectra were recorded by a microplate reader (PerkinElmer, USA).

Preparation of ETNs

The erythrocytes were collected from anticoagulant whole blood and then fixed with 4% paraformaldehyde (PFA) for 24 h, after that, the erythrocytes were transferred into 1 M NaCl and dried after fully mixed using Vacuum freeze-drying apparatus. After high-temperature calcination (800℃) of the as-obtained precursor and following washed with ultrapure water, ETN was obtained, at the same time, the fixed erythrocytes untreated with NaCl, and ETNs obtained from different carbonization temperature were used as controls.

Peroxidase activity and kinetic parameters analysis.

The enzyme activity selection of various carbon nanozymes were performed by comparing the peroxidase activity and kinetic parameters using the method as described previously.[1] For details, the peroxidase-like activity assays of ETN were carried out using TMB (in DMSO, 20 μL, 10 mg mL^{-1}, Sigma Aldrich) as the substrate in the presence of H_2O_2 (15 μL, 10 M, Sinopharm Chemical Reagent Co., Ltd.) in sodium acetate buffer (1 mL, 0.1 M, pH 4.5). The absorbance of the chromogenic reactions (652 nm for TMB) represented the peroxidase-like activity was recorded at certain reaction times via a microplate reader (PerkinElmer, USA).

The peroxidase-like activity of the pH dependence of ETN was performed in different buffer solutions with pH values from 2 to 11, and the temperature dependence was performed in different temperatures increased gradually from 25℃ to 90℃.

The steady-state kinetic assays were carried out at 37°C in sodium acetate solution (NaAc, 1 mL, 0.2 M, pH 4.5) with ETN (10 μL, 1 mg mL^{-1}) in the presence of H_2O_2 and TMB. The kinetic assays of ETN with TMB as the substrate were performed

by adding H_2O_2 (15 μL, 10 M) and different amounts of TMB (in DMSO, 10 mg mL^{-1}, 2, 4, 8, 15, 25, 40, 50 μL). The kinetic assays of ETN with H_2O_2 as the substrate were performed by adding TMB (Dissolved in DMSO, 20 μL, 10 mg mL^{-1}) and different amounts of H_2O_2 (10 M, 2, 4, 8, 15, 30, 60, 120 μL). The Michaelis-Menten constants were calculated according to the Michaelis - Menten saturation curve by GraphPad Prism 8 (GraphPad Software). For comparison, the peroxidase-like activity of ETN without NaCl was also measured under the same conditions.

The OXD-like activity of ETN and ETN without NaCl were evaluated using TMB (in DMSO, 50 μL, 10 mg/mL, Sigma Aldrich) as the substrate in NaAc buffer (1 mL, 0.1 M, pH 4.5). The absorbance at the 652 nm (oxTMB) was recorded at certain reaction times via the Victor Nivo™ Multimode Plate Reader (PerkinElmer, USA).

The SOD-like activity of ETN was evaluated using a SOD assay kit (Dojindo Laboratories, Japan). Firstly, different concentrations (0–1 mg mL^{-1}) of ETN was mixed with 200.0 μL WST-1 working solution. Then, 20 μL of xanthine oxidase solution was added to initiate the reaction. After incubating at 37℃ for 20 min, SOD-like activity was obtained by quantifying the decrease at 450 nm.

The CAT-like activity of ETN was measured by using a specific oxygen electrode on a multi-parameter analyzer (JPSJ-606L, Leici China) to monitor the increase in dissolved O_2 concentration. The ETN (2 μg mL^{-1}) mixed with H_2O_2 (500 mM) aqueous solution. The reaction was conducted in the total volume of 5 mL. All reactions were carried out in water at 37℃.

Temperature changes of ETN under NIR irradiation

ETN with different concentration (0, 125, 250, 500, 1000 μg mL^{-1} in NaAC) was combined with 100 μM of H_2O_2, and temperature was recorded by a thermometer under 808 nm laser irradiation (2 W cm^{-2}). The temperature of NaAC, H_2O_2 (100 μM), ETN (500 μg mL^{-1}) and ETN + H_2O_2 under 808 nm laser irradiation (2 W cm^{-2}, 6 min) were also recorded.[2]

Antibacterial activity of ETN *in vitro*

Gram-positive methicillin-resistant *S. aureus* (MRSA, ATCC43300), *S. aureus* (ATCC 29213) and Gram-negative bacteria *E. coli* (CMCC (B)44102) was used for antibacterial assay. The MRSA was cultured in the standard LB (Luria-Bertani) medium. The ETNs were placed in the ep pipes, the MRSA (1 mL, 1 × 10^6 CFU mL^{-1}) was co-incubated with NaAc buffer, H_2O_2 (100 μM in NaAC), NaAC + NIR, ETN, H_2O_2 + ETN (500 μg mL^{-1} in NaAC), ETN + H_2O_2 + NIR, ETN + NIR, H_2O_2 + NIR, respectively. the (NIR+) samples were irradiated with the 808 nm NIR laser (2 W cm ^{2}) for 6 min.

Standard Plate Counting Assays: After incubation, the dissociated bacteria suspension was diluted 10^5 times for bacterial with PBS. The diluted bacteria suspension (100 μL) of different samples plated on the standard LB agar via spread plate method and cultivated another 24 h at 37℃. The lived bacteria were counted, and the antibacterial effect was calculated.

Morphology observation by SEM: To further confirm antibacterial effect of ETNs, the integrity of bacterial membrane was further checked by SEM. 2.5% glutaraldehyde solution was used to examine the morphology of the bacteria on

different samples. Afterwards, the samples were rinsed with sterile PBS twice and serially dehydrated using a series of ethanol solutions (50, 70, 80, 90, 95 and 100% v/v) for 14 min sequentially. Finally, all samples were dried and covered with gold for SEM observation.

Total ROS measurement. MRSA was diluted at 1:1000 and cultured until OD600 reached 0.8, 1 mL of bacteria was centrifuged at 3600 rpm for 5 min at 4°C, and the precipitate was diluted with 100 μL H_2O. The groups as follows: (1) Control; (2) NaAC + NIR; (3) H_2O_2 + NIR; (4) H_2O_2; (5) ETN + NIR; (6) ETN; (7) ETN + H_2O_2 + NIR; (8) ETN + H_2O_2. (ETN and H_2O_2 were diluted into 500 μg mL^{-1} and 100 μM by NaAC, respectively) for different treatments. After that, the samples were centrifuged at 3600 rpm for 5 min at 4°C. DCFH-DA was 1:1000 diluted with medium to the action concentration of 10 μM. After the resuspended bacterial solution was precipitated, it was incubated at 37℃ for 30 min in the dark, and then upside-down every 5 minute.[3] The samples were washed twice with normal saline, and the total ROS level was measured by excitation at 488 nm and emission at 525 nm.

Live and dead staining test. The MRSA were treated with ETN or different conditions, and then stained with SYTO9 green fluorescent nucleic acid stain (5 μM) for 20 min and washed twice with normal saline. Then the bacteria incubated with propidium iodide (PI, 1 μg mL^{-1}) for another 20 min in the dark, then washed with normal saline twice. Next, the samples were washed twice with PBS before being analyzed using laser confocal microscope LSM700 (ZEISS, Germany). Glutathione levels were measured using GSH assay kit (Beyotime, China).

Biosafety evaluation of ETN

Cytotoxicity assay: The Raw264.7 (ATCC) and HaCaT cells (ATCC) were plated in 96-well plates with 5×10^4 cells per well. After 12 h of growth, cells were treated with different concentrations of ETN for 24 h and 10 μL CCK-8 was then added. After 1 h, the medium supernatant was taken to read 450 nm.

Hemolysis test: 1 mL of fresh blood was added with 2 mL PBS and centrifuged at 10000 g for 5 min, then discard the supernatant and added 10 mL PBS. Taken 0.1 mL suspended solution added with 0.4 mL ETN working solution of different concentrations as test groups, 0.4 mL H_2O was added as the positive control group, 0.4 mL PBS was used as the negative control group. After 4 h, all groups were centrifuged at 10000 g for 5 min. Taken 100 μL sample and measured absorbance at 577 nm. Hemolysis rate % = (sample OD-negative OD) / (positive OD - negative OD) × 100%.

Blood clotting tests of ETN *in vitro*

90 μL blood was mixed with 5 μL calcium chloride (0.2 M) and then added 5 μL ETN (different concentration dispersed in water), then the mixture was stored at 37°C for 5 min and whereafter centrifuge at a low speed of 500 rpm for 3 minutes. After that, 1 mL water was slowly added without blowing, take photos and measure the absorption of supernatant at 575 nm.[4]

Western Blot of Fibrinogen and prothrombin

Rabbit anticoagulant whole blood (sodium citrate anticoagulant, Bersee) was taken

and centrifuged at 4000 rpm for 10 min at 4℃. After centrifugation, the supernatant was collected, which was rabbit plasma. The collected rabbit plasma was divided into EP tubes on average, and 5 μL different concentrations (0, 62.5, 125, 500 μg mL^{-1}) of ETN were added into 90 μL rabbit plasma, the mixture was incubated at 37℃ for 5 min. Finally, the samples were centrifuged at 12000 rpm for 5 min at 4℃, and the precipitates were collected after the centrifugation to obtain the pre-treated samples. The resulting samples were used in subsequent Western Blot experiments.[5] The antibodies were Anti-Fibrinogen (Abcam, ab118488) and Anti-Prothrombin (Abcam, ab109087), both diluted at 1:1000.

Procoagulant effect of ETN in vivo

Before the experiment, cotton balls were socked with 0.5 mg mL^{-1} ETN (dispersed in sodium acetate solution (1 mL, 0.1 M, pH 4.5), cotton balls socked with sodium acetate solution and cotton balls socked with water as controls. For the procoagulant effect in vivo, 8-week-old wild type balb/c mice were divided into three groups as follows: Control group, buffer group and ETN group. After the mice were anesthetized, part of the liver tissue was removed, cotton balls of the above three treatments were placed to compress the wound until the bleeding stopped. The bleeding time and the weight of the removed liver were recorded, at the same time, the weight of cotton balls of mice were sutured after hemostasis.

Antibacterial activity and wound healing *in vivo*

All animal studies were performed following the protocols approved by the Institutional Animal Care and Use Committee of Institutional Animal Care and Use Committee of the Institute of Biophysics, Chinese Academy of Sciences. Female balb/c mice (age: 8-10 weeks) were obtained from Vital River Laboratories.

The backs of the mice were shaved and anesthetized with isoflurane. A round full-thickness skin wound (Φ10 mm) was created on the back of each mouse through a hole punch. 30 μL MRSA mixed suspension (5×10^7 CFU) was injected into the center of each wound. Mice were arbitrarily divided into eight groups (n = 6).[6] Followed as: (1) control; (2) NaAC + NIR; (3) H_2O_2 + NIR; (4) H_2O_2; (5) ETN + NIR; (6) NIR; (7) ETN + H_2O_2 + NIR; (8) ETN + H_2O_2. After 24 h, for the ETN + H_2O_2 + NIR group, a total of 20 μL (ETN 500 μg mL^{-1} and H_2O_2 100 μM, all dispersed into NaAC) were dripped on the wound, then illuminated with an 808 nm laser (2 W cm^{-2}) for 6 min. The other seven groups used the similar method. NaAC were added at the same time as laser irradiation to avoid scorching. The wounds of each group were photographed every day and the wound area were measured. In addition, to check the antibacterial activity *in vivo*, on the 7th day, the wound skin tissue was grinded and diluted to varying degrees with 1 mL LB, and added 100 μL diluent into the agar culture plate and incubate at 37℃ for 18 h, the antimicrobial properties *in vivo* were assessed by CFUs. In addition, the heart, liver, spleen, lung and kidney of all mice were removed for H&E staining.

Statistical Analysis

All data were expressed as mean ± standard error of mean (SEM) and subjected to analysis using GraphPad Prism 8 (GraphPad Software, San Diego, CA, USA). Multiple

group comparisons were performed using one-way analysis of variance test (ANOVA) with Fisher's least significant difference (LSD) *post hoc* test, and while two samples group comparisons were conducted were compared by using unpaired two-tailed Student's *t* test. Statistical significance of the data in the present study was expressed as $*p < 0.05$, $**p < 0.01$, $***p < 0.001$, and $****p < 0.0001$.

References

[1] Q. Liang, J. Xi, X. J. Gao, R. Zhang, Y. Yang, X. Gao, X. Yan, L. Gao, K. Fan, *Nano Today* 2020, 35, 100935.
[4] D. Xi, M. Xiao, J. Cao, L. Zhao, N. Xu, S. Long, J. Fan, K. Shao, W. Sun, X. Yan, X. Peng, *Adv Mater* 2020, 32, e1907855.
[6] R. Ma, L. Fang, L. Chen, X. Wang, J. Jiang, L. Gao, *Theranostics* 2022, 12, 2266.
[2] Q. Xia, Z. Liu, C. Wang, Z. Zhang, S. Xu, C. C. Han, *Biomacromolecules* 2015, 16, 3083.
[3] a)C. Oslakovic, T. Cedervall, S. Linse, B. Dahlback, *Nanomedicine* 2012, 8, 981; b)V. Gryshchuk, N. Galagan, *Biochem Res Int* 2016, 2016, 2959414; c)C. Li, M. Hu, S. Jiang, Z. Liang, J. Wang, Z. Liu, H. D. Wang, W. Kang, *Molecules* 2020, 25, 177.
[5] Y. Liu, X. Wang, X. Fan, M. Ge, L. Fang, Y. Yuan, L. Chen, J. Jiang, A. Cao, L. Gao, *Advanced Functional Materials* 2023, 33, 2212655.
